# A new species of *Neoperla* from China, with a redescription of the female of *N. mnong* Stark, 1987 (Plecoptera, Perlidae)

**DOI:** 10.3897/zookeys.616.8996

**Published:** 2016-09-12

**Authors:** Zhi-Teng Chen, Yu-Zhou Du

**Affiliations:** 1 School of Horticulture and Plant Protection & Institute of Applied Entomology, Yangzhou University, Yangzhou 225009, China

**Keywords:** China, Neoperla, new species, Perlidae, Plecoptera

## Abstract

A new species of the *Neoperla clymene* group (Plecoptera, Perlidae), *Neoperla chebalinga* **sp. n.** from Guangdong Province of southern China is described, illustrated, and compared with related taxa. The new species is characterized by the slender aedeagal tube, strongly sclerotized dorsally, and weakly sclerotized ventrally with an upcurved, medial, finger-like membranous lobe. Additionally the aedeagal sac gradually tapers to a blunt apex with a dorsoapical patch of spines. A supplementary description of the female of *Neoperla mnong* Stark, 1987 from Guangdong Province, China is also given.

## Introduction

*Neoperla* Needham (1905) is the most species-rich perlid genus in China with at least 57 species (DeWalt et al. 2016). Contributions to Chinese species of *Neoperla* were made by Chu (1929), Wu and Claassen (1934), Wu (1935, 1938, 1948, 1962, 1973), Yang and Yang (1992, 1995, 1998), Du (1998, 1999, 2000a, 2000b), Du et al. (1999, 2001), Du and Sivec (2004, 2005), Du and Wang (2005, 2007), Sivec and Zwick (1987), Li and Wang (2011), Li et al. (2011a, 2011b, 2012a, 2012b, 2013a, 2013b, 2014a, 2014b), Qin et al. (2013), Wang et al. (2013a, 2013b), Li and Zhang (2014), Kong et al. (2014), Wang et al. (2014), Chen and Du (2015, 2016). Herein, a new species of this genus is described from Guangdong Province, a coastal region of southern China. Additionally, a supplementary description and new illustrations for the female of *Neoperla mnong* Stark, 1987 from Guangdong Province are provided to indicate the variation of the female subgenital plate and head pattern as compared to the original description of Vietnamese material.

## Material and methods

Specimens used in this study were collected from riparian areas by hand and preserved in 75% ethanol. Abdomens were cleared in 10% NaOH. Details of the morphology were studied with a Leica MZAPO microscope, and color illustrations were taken by Leica SZ45 and S4800 FESEM, both in Yangzhou, Jiangsu Province, China. All studied material, including the holotype and paratypes of the new species, are deposited in the Insect Collection of Yangzhou University, China.

## Taxonomy

### Neoperla chebalinga sp. n.

Taxon classificationAnimaliaPlecopteraPerlidae

http://zoobank.org/960227BF-2234-483D-9363-3C7A40D0F32D

Figs 1 , 2–3 , 4–5 , 6 , 7 , 8 , 9–10

#### Type material.

Holotype male: China: Guangdong Province, Chebaling Nature Reserve, 114.1320E, 24.7128N, 17 June, 2009, leg. Hai-Yang Xue and Bo Yu. Paratypes: 4 males and 2 females, same data as holotype.

#### Diagnosis.

Distinguishing characteristics of this species include a slender aedeagal tube that has an upcurved, medial, finger-like, membranous lobe on the ventral surface, and a short, blunt, ventrally curved aedeagal sac with a dorsoapical patch of spines.

**Figure 1.**
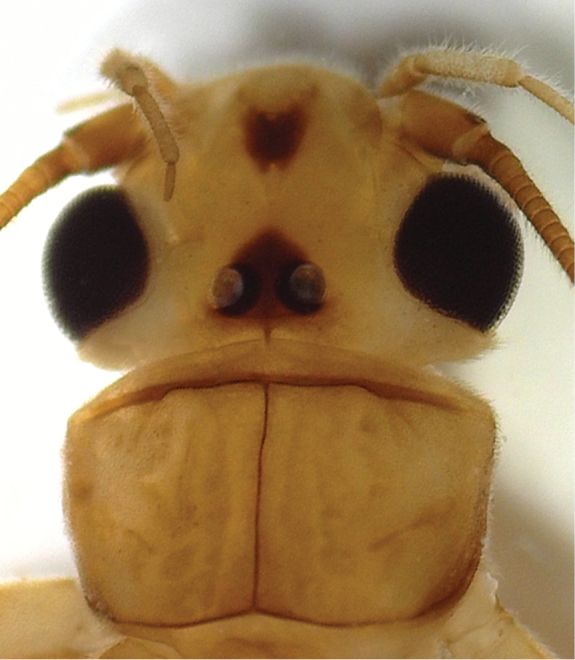
*Neoperla chebalinga* sp. n. Male head and pronotum, dorsal view.

**Adult habitus.** General body color yellow patterned with brown. Head slightly wider than pronotum with a brown stigma on anterior frons and a subtriangular dark brown area covering ocelli; compound eyes dark and antennae brown (Figs 1, 9–10). Pronotum disc yellow with pale brown rugosities (Fig. 1). Wing membrane subhyaline, veins brown; legs yellow, femora grading to brown distally. Cerci pale (Figs 9–10).

**Figures 2–3.**
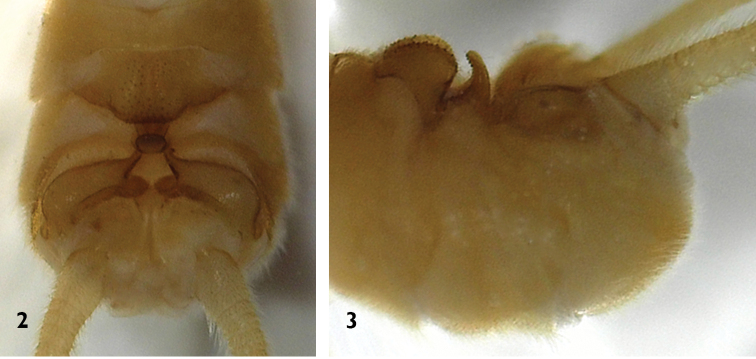
*Neoperla chebalinga* sp. n. Male terminalia: **2** dorsal view **3** lateral view.

**Figure 4–5.**
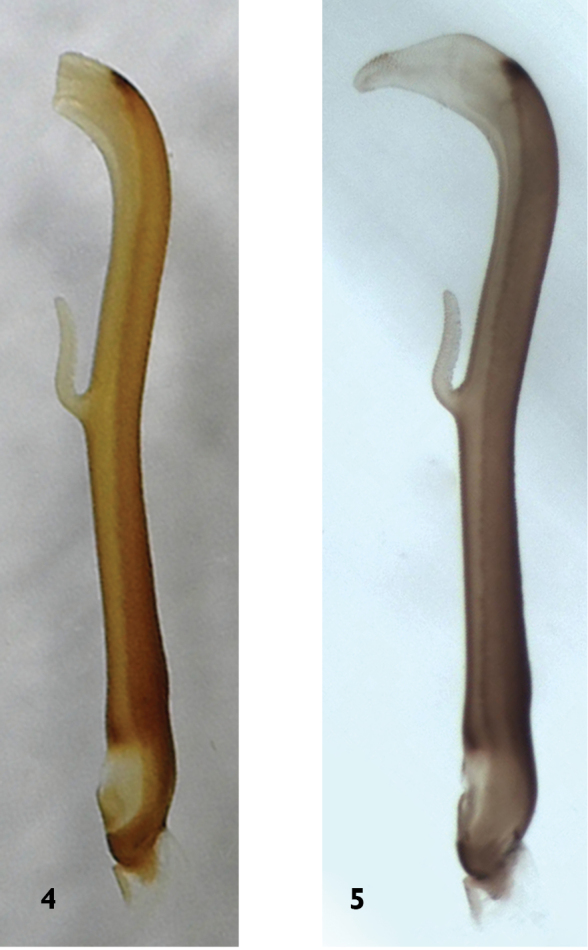
*Neoperla chebalinga* sp. n. Aedeagus: **4** *In situ*, lateral view **5** Everted, lateral view.

**Male** (Figs 1–5, 9). Forewing length 13.5–14.0 mm, hindwing length 11.5–12.0 mm (n = 5). The posterior margin of tergum 7 with a raised process densely covered with sensilla basiconica. Tergum 8 bears a recurved oval process with small spines at the distal margin (Figs 2–3). Tergum 9 without sensilla basiconica. Hemitergal processes of tergum 10 slightly curved anteriorly (Fig. 2). Aedeagal tube slender, strongly sclerotized dorsally, weakly sclerotized ventrally except for the basal bulb and an upcurved, ventromedial, finger-like membranous lobe. Aedeagal sac short and gradually tapered to a blunt apex, curved ventrally with a dorsoapical patch of spines (Figs 4–5).

**Figure 6.**
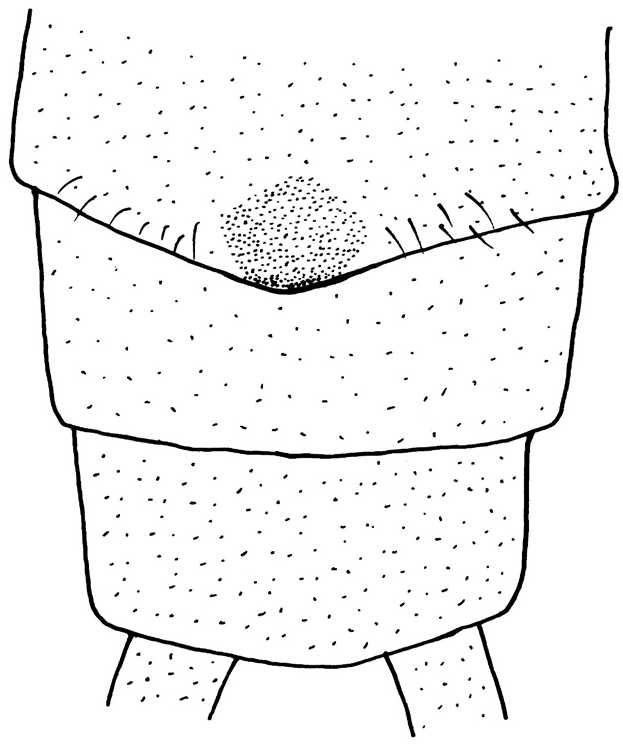
*Neoperla chebalinga* sp. n. Female terminalia, ventral view.

**Figure 7.**
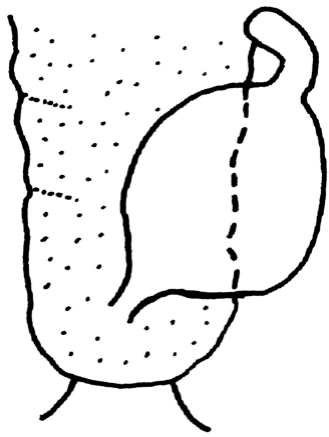
*Neoperla chebalinga*, sp. n. Female genitalia, vagina and spermathecum, lateral view.

**Female** (Figs 6–7, 10). Forewing length 15.5–16.0 mm, hindwing length 13.0–13.5 mm (n = 2). General color and pattern similar to males. Subgenital plate of sternum 8 slightly produced and lightly sclerotized posteromedially (Figs 6, 10). Vagina elongate-oval, lined sparsely along lateral margins. Spermathecal stalk short, spermatheca oval and curled at tip (Fig. 7).

**Figure 8.**
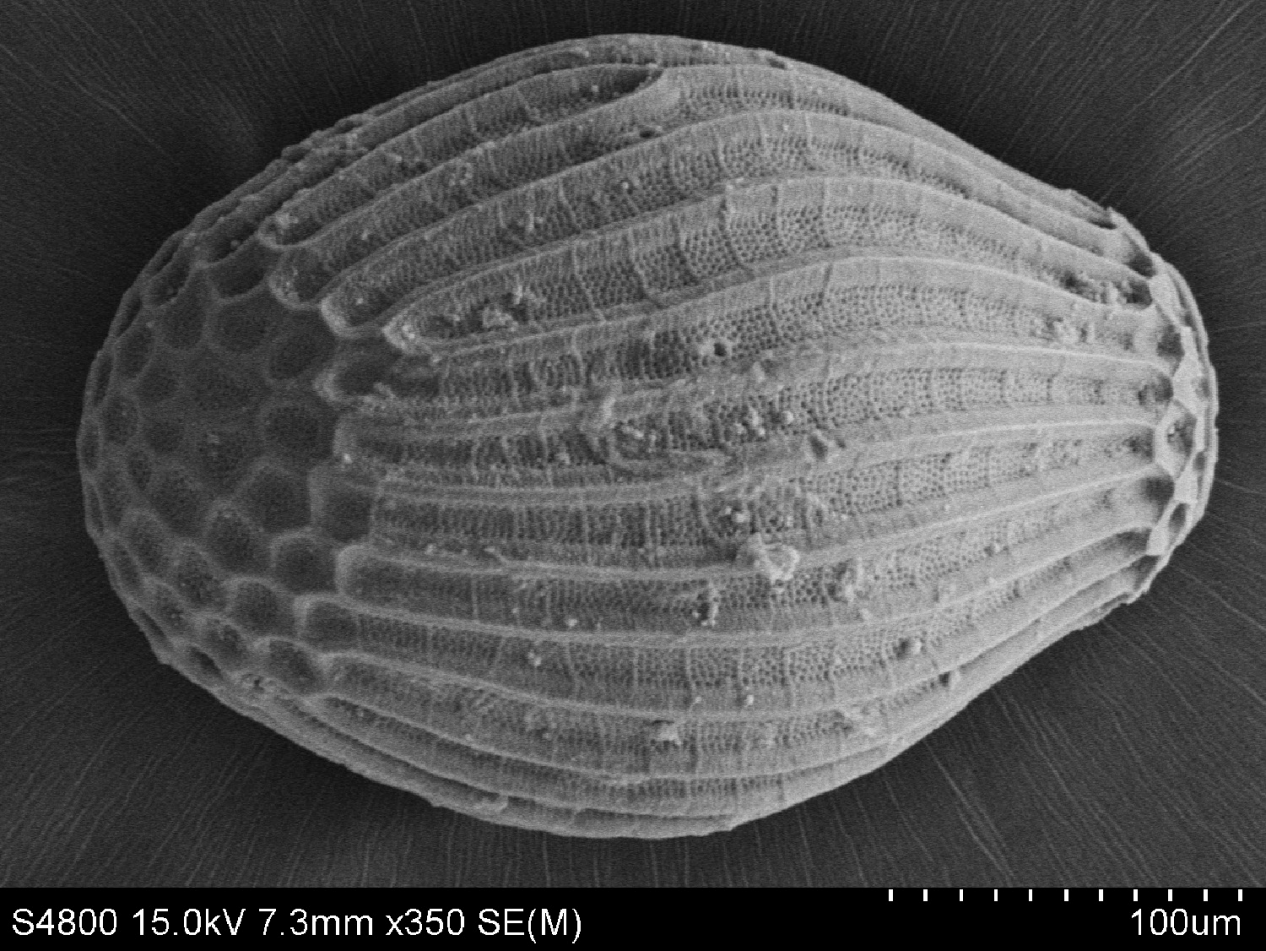
*Neoperla chebalinga* sp. n. Egg, lateral view.

**Eggs** (Fig. 8). Outline oval. Collar short and surrounded by 1–2 irregular rows of follicle cell impressions. Primary striae join rim on sides of collar, narrow and widely spaced; each pair of primary striae enclose many secondary striae; sulci with dense pits. Lid small consisting of follicle cell impressions (Fig. 8).

**Figure 9–10.**
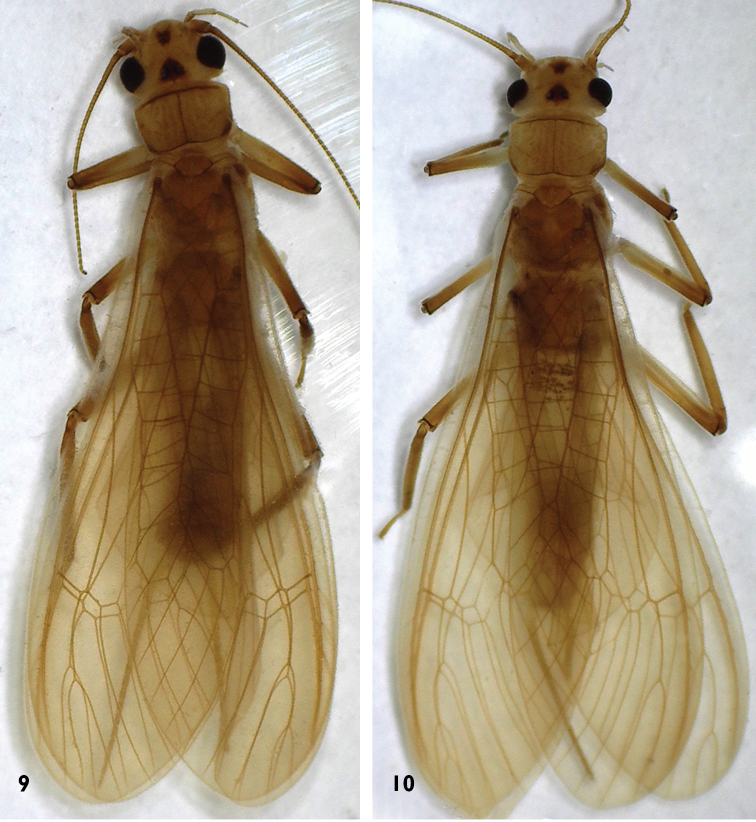
*Neoperla chebalinga* sp. n. Habitus: **9** Male habitus, dorsal view **10** Female habitus, dorsal view.

#### Etymology.

The species is named after the type locality, Chebaling Nature Reserve.

#### Remarks.

The new species is a member of the *Neoperla clymene* group possessing a slender and mostly sclerotized aedeagal tube. The processes of terga 7–8 and aedeagus of the new species are similar to those of *Neoperla furcostyla* Li & Qin, 2013 (in: Li et al. 2013b) and *Neoperla forcipata* Yang & Yang, 1992 (Yang and Yang 1992). However, in *Neoperla furcostyla* and *Neoperla forcipata*, the aedeagal tube has a subapical fork instead of the medial finger-like lobe as in *Neoperla chebalinga*. In addition, the aedeagal sacs of *Neoperla furcostyla* and *Neoperla forcipata* are not curved ventrally (see Figs 1–2 in: Li et al. 2013b and fig. 1 in Yang and Yang 1992).

### Neoperla mnong

Taxon classificationAnimaliaPlecopteraPerlidae

Stark, 1987

Figs 11 , 12 , 13

Neoperla mnong Stark, 1987: 48; Stark & Sivec 2008: 33; Du 1998: 393; Li et al. 2012: 22; Wang et al. 2013b: 87.

#### Material examined.

1 male, 4 females, China: Guangdong Province, Chebaling Nature Reserve, 114.1320E, 24.7128N, 17 June, 2009, leg. Hai-Yang Xue and Bo Yu.

#### Description of the female Chinese specimens.

**Figure 11.**
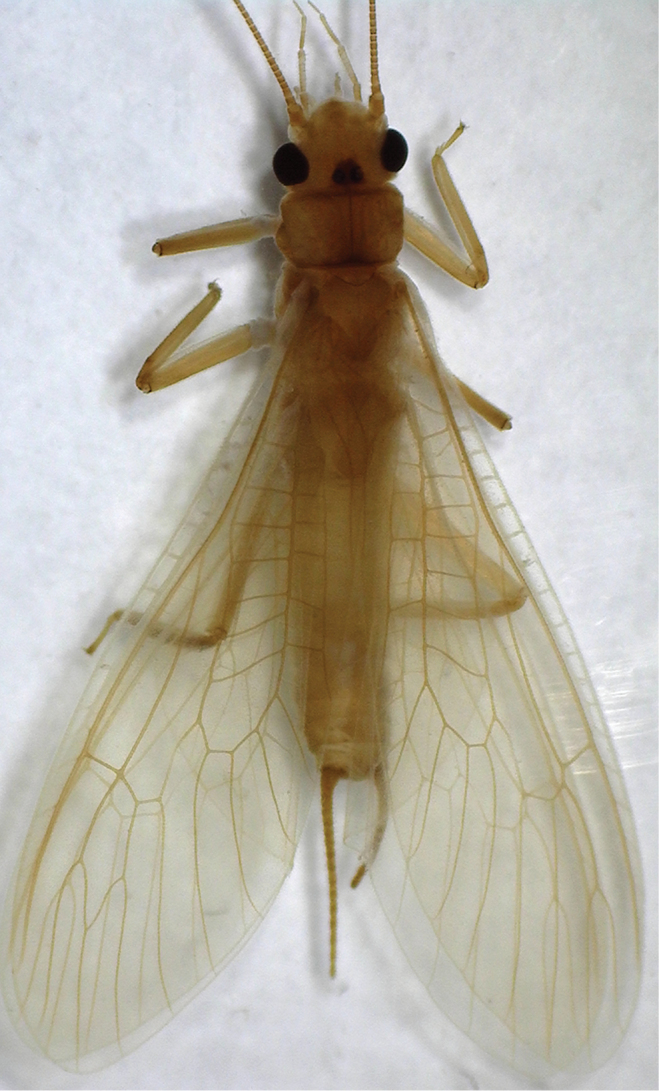
*Neoperla mnong* Stark. Female habitus, dorsal view.

**Figure 12.**
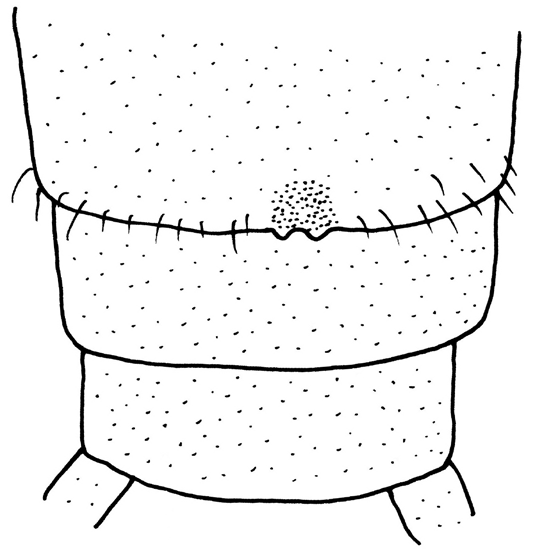
*Neoperla mnong* Stark. Female terminalia, ventral view.

**Figure 13.**
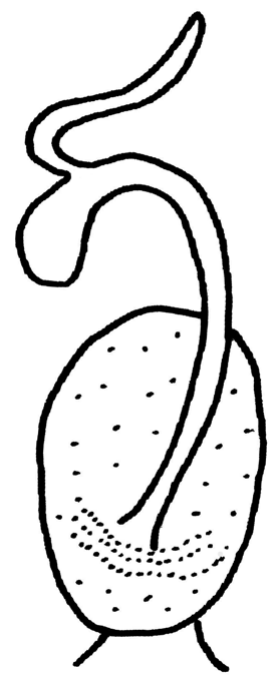
*Neoperla mnong* Stark. Female genitalia, vagina and spermathecum, lateral view.

**Female** (Figs 11–13). Forewing length 12.0–12.5 mm, hindwing length 10.0–10.5 mm (n = 4). General color yellow to brown. Head nearly as wide as pronotum, with a subtriangular dark brown area covering ocelli; compound eyes dark and antennae pale brown. Pronotum disc yellow with pale brown rugosities. Wing membrane subhyaline, veins pale brown; legs yellow without darker markings. Cerci pale brown (Fig. 11). Subgenital plate of sternum 8 forming a small rounded sclerite with a posteromedial notch (Fig. 12). Vagina oval, lined densely around base of spermathecal stalk. Spermathecal stalk and spermatheca short, slender and curled at tip; a single accessory gland occurs near spermatheca tip (Fig. 13).

#### Distribution.

China (Guangdong Province: Conghua County, Liuxi River; Shixing County, Chebaling Nature Reserve. Guangxi Province: Jinxiu suburb; Tianlin County, Pinglang). Vietnam (Di Linh, Dak Song, Gia Lai, Nghe An, Pleiku). Thailand (Chanthaburia).

#### Remarks.

This species is expected to be widespread in southern Asia. The male of *Neoperla mnong* has been described from Vietnam by Stark (1987) and well-illustrated by Stark and Sivec (2008) from Vietnamese material. This species has been previously recorded from China by Du (1998) and Li et al. (2012b). Females of *Neoperla mnong* from Vietnam were described as *Javanita costalis* by Navás (1932) and later described by Stark and Sivec (2008). In this study, females collected from Guangdong Province of China in 2009 are described and illustrated for the first time. The subgenital plate of these specimens is smaller and pigment patch over the ocelli is larger (for comparison see Figs 40, 43 in Stark and Sivec 2008).

## Supplementary Material

XML Treatment for Neoperla chebalinga

XML Treatment for Neoperla mnong
